# Supplementary material for: Early Life Factors and Polycystic Ovary Syndrome in a Swedish Birth Cohort
Source: Int J Environ Res Public Health. 2023 Nov 20;20(22):7083. doi: 10.3390/ijerph20227083 (PMC10671095; doi:10.3390/ijerph20227083)

International Journal of *Environmental Research and Public Health*

MDPI

Table S1. Representation of modelling strategy

**Full sample: 1973-1995**						
	**Model 1**	**Model 2**	**Model 3**	**Model 4**		**Model 5**
Intergenerational sociodemographic factors	MA	Group	X	X		X
Intergenerational biological variables	MA	Group	X	X		X
Early-life factors: outcomes	MA	Group		X		X
Individual adult life factors	MA	Group				X

**Restricted sample: 1982-1995**						
	**Model 1**	**Model 2**	**Model 3**	**Model 4a**	**Model 4b**	**Model 5**
Intergenerational sociodemographic factors	MA	Group	X	X	X	X
Intergenerational biological variables	MA	Group	X	X	X	X
Early-life factors: before birth	MA	Group		X	X	X
Early-life factors: outcomes	MA	Group			X	X
Individual adult life factors	MA	Group				X

Abbreviations: MA, minimally adjusted

Table S2. Associations of social and biological characteristics with rate of polycystic ovary syndrome among Swedish women born between 1973-1995

Predictor	Model 1 [Full] HR CI 95%	Model 1 [Res.] HR CI 95%	Model 2 [Full] HR CI 95%	Model 2 [Res.] HR CI 95%	Model 3 [Full] HR CI 95%	Model 3 [Res.] HR CI 95%	Model 4 [Full] HR CI 95%	Model 4a [Res.] HR CI 95%	Model 4b [Res.] HR CI 95%	Model 5 [Full] HR CI 95%	Model 5 [Res.] HR CI 95%
**Intergenerational sociodemographic factors**											
Birth order											
First born **[Ref]**	**1*****	**1*****	**1*****	**1*****	**1*****	**1*****	**1*****	**1*****	**1*****	**1****	**1*****
Second born	0.911	0.875	0.915	0.876	0.915	0.870	0.922	0.864	0.872	0.924	0.873
	[0.873 - 0.951]	[0.811 - 0.943]	[0.877 - 0.956]	[0.812 - 0.946]	[0.876 - 0.955]	[0.805 - 0.939]	[0.882 - 0.963]	[0.780 - 0.933]	[0.807 - 0.943]	[0.884 - 0.965]	[0.809 - 0.945]
Third born or higher	0.998	1.072	0.965	1.036	0.951	1.020	0.959	0.997	1.007	0.957	1.000
	[0.940- 1.061]	[0.981- 1.173]	[0.908 - 1.027]	[0.945 - 1.136]	[0.893 - 1.014]	[0.927 - 1.121]	[0.900 - 1.022]	[0.906 - 1.097]	[0.914 - 1.109]	[0.898 - 1.020]	[0.908 - 1.102]
Mother´s age at index woman´s birth, years											
Less than or equal to 18	1.220	1.204	1.128	1.036	1.126	1.035	1.122	1.054	1.058	1.118	1.046
	[1.054 - 1.413]	[0.845 - 1.715]	[0.973 - 1.307]	[0.742 - 1.498]	[0.972 - 1.305]	[0.724 - 1.478]	[0.968 - 1.300]	[0.737 - 1.506]	[0.740 - 1.512]	[0.964 - 1.296]	[0.732 - 1.496]
Between 19-35 **[Ref]**	**1****	**1***	**1***	**1**	**1***	**1**	**1***	**1**	**1**	**1***	**1**
Greater than 35	1.066	1.048	1.065	1.049	1.047	1.024	1.047	1.009	1.008	1.036	1.000
	[0.997 - 1.139]	[0.935 - 1.175]	[0.996 - 1.139]	[0.934 - 1.179]	[0.978 - 1.119]	[0.911 - 1.151]	[0.979 - 1.121]	[0.897 - 1.134]	[0.896 - 1.133]	[0.969 - 1.109]	[0.890 - 1.125]
Mother´s educational attainment											
Primary	1.071	1.100	0.991	0.992	0.984	0.984	0.983	0.959	0.957	0.987	0.955
	[1.017 - 1.127]	[0.995 - 1.217]	[0.940 - 1.046]	[0.894 - 1.101]	[0.933 - 1.038]	[0.887 - 1.093]	[0.933 - 1.037]	[0.864 - 1.065]	[0.863 - 1.064]	[0.935 - 1.041]	[0.860 - 1.061]
Secondary **[Ref]**	**1*****	**1*****	**1**	**1***	**1**	**1***	**1**	**1**	**1**	**1**	**1**
University	0.941	0.884	0.975	0.937	0.982	0.945	0.983	0.982	0.984	0.981	0.981
	[0.904 - 0.981]	[0.821 - 0.952]	[0.932 - 1.020]	[0.864 - 1.017]	[0.938 - 1.028]	[0.871 - 1.026]	[0.942 - 1.031]	[0.904 - 1.066]	[0.905 - 1.068]	[0.937 - 1.027]	[0.903 - 1.065]
Father´s educational attainment											
Primary	1.042	1.022	1.011	0.983	1.004	0.979	1.005	0.964	0.965	1.018	0.975
	[0.997 - 1.090]	[0.940 - 1.111]	[0.966 - 1.059]	[0.903 - 1.071]	[0.959 - 1.051]	[0.890 - 1.066]	[0.964 - 1.056]	[0.885 - 1.049]	[0.886 - 1.050]	[0.972 - 1.066]	[0.895 - 1.061]
Secondary **[Ref]**	**1*****	**1*****	**1**	**1***	**1**	**1***	**1**	**1***	**1***	**1***	**1 ***

University	0.945	0.871	0.981	0.920	0.984	0.924	0.985	0.951	0.952	0.977	0.948
	[0.905 - 0.988]	[0.804 - 0.945]	[0.934 - 1.031]	[0.841 - 1.007]	[0.937 - 1.034]	[0.844 - 1.011]	[0.939 - 1.036]	[0.869 - 1.042]	[0.869 - 1.043]	[0.930 - 1.026]	[0.865 - 1.039]
Mother´s country of birth											
Sweden **[Ref]**	**1*** **	**1*** **	**1*** **	**1 *** **	**1*** **	**1 *** **	**1*** **	**1*** **	**1*** **	**1*** **	**1*** **
Nordics, excluding Sweden	1.062	1.035	1.017	0.994	1.017	0.992	1.018	0.975	0.974	0.988	0.949
	[0.972 - 1.160]	[0.873 - 1.226]	[0.926 - 1.117]	[0.833 - 1.186]	[0.926 - 1.117]	[0.832 - 1.184]	[0.927 - 1.118]	[0.817 - 1.163]	[0.816 - 1.162]	[0.900 - 1.086]	[0.795 - 1.134]
Europe, North America and	1.573	1.632	1.366	1.423	1.350	1.413	1.353	1.444	1.441	1.299	1.371
Oceania	[1.446 - 1.712]	[1.399 - 1.903]	[1.235 - 1.511]	[1.184 - 1.711]	[1.220 - 1.495]	[1.175 - 1.700]	[1.223 - 1.497]	[1.200 - 1.737]	[1.198 - 1.734]	[1.173 - 1.438]	[1.138 - 1.652]
Africa	1.796	2.115	1.291	1.490	1.274	1.475	1.271	1.542	1.541	1.211	1.471
	[1.465 - 2.202]	[1.515 – 2.953]	[0.996 - 1.675]	[0.968 - 2.295]	[0.982 - 1.653]	[0.956 - 2.274]	[0.979 - 1.649]	[1.001 - 2.374]	[1.001 - 2.374]	[0.933 - 1.572]	[0.954 - 2.270]
Asia	2.418	2.215	1.658	1.549	1.608	1.506	1.604	1.634	1.626	1.553	1.599
	[2.140 - 2.732]	[1.785 - 2.750]	[1.402 - 1.960]	[1.156 - 2.075]	[1.358 - 1.903]	[1.123 – 2.019]	[1.355 - 1.898]	[1.218 - 2.193]	[1.211 - 2.183]	[1.313 - 1.838]	[1.191 - 2.146]
South America	1.960	1.648	1.579	1.143	1.564	1.134	1.566	1.159	1.263	1.480	1.100
	[1.573 - 2.443]	[1.112 – 2.443]	[1.187 - 2.100]	[0.699 - 1.867]	[1.176 - 2.082]	[0.693 - 1.856]	[1.176 - 2.084]	[0.708 - 1.898]	[0.711 - 1.903]	[1.113 - 1.968]	[0.672 - 1.798]
Father´s country of birth											
Sweden **[Ref]**	**1*** **	**1*** **	**1*** **	**1***	**1*** **	**1***	**1*** **	**1***	**1***	**1*** **	**1***
Nordics, excluding Sweden	1.091	1.070	1.060	1.033	1.055	1.020	1.055	1.008	1.010	1.047	1.005
	[0.991 - 1.201]	[0.892 - 1.283]	[0.958 - 1.175]	[0.855 - 1.249]	[0.953 - 1.168]	[0.843 - 1.232]	[0.952 - 1.168]	[0.834 - 1.219]	[0.834 - 1.218]	[0.945 - 1.160]	[0.831 - 1.215]
Europe, North America and	1.444	1.445	1.196	1.142	1.187	1.129	1.186	1.140	1.137	1.134	1.091
Oceania	[1.334 - 1.563]	[1.245 - 1.677]	[1.087 - 1.315]	[0.954 - 1.366]	[1.079 - 1.306]	[0.943 - 1.353]	[1.078 - 1.304]	[0.952 - 1.366]	[0.949 - 1.362]	[1.030 - 1.248]	[0.910 - 1.310]
Africa	1.677	1.809	1.362	1.306	1.335	1.268	1.333	1.257	1.251	1.237	1.144
	[1.422 - 1.978]	[1.364 - 2.340]	[1.103 - 1.682]	[0.905 - 1.884]	[1.081 - 1.649]	[0.878 - 1.830]	[1.079 - 1.647]	[0.872 - 1.812]	[0.867 - 1.804]	[1.001 - 1.528]	[0.792 - 1.652]
Asia	2.374	2.199	1.624	1.508	1.594	1.466	1.593	1.440	1.435	1.516	1.366
	[2.109 - 2.672]	[1.786 - 2.707]	[1.379 - 1.912]	[1.135 - 2.005]	[1.353 - 1.879]	[1.102 - 1.950]	[1.352 - 1.878]	[1.082 - 1.915]	[1.079 - 1.910]	[1.287 - 1.787]	[1.027 - 1.818]
South America	1.762	1.822	1.313	1.584	1.306	1.552	1.306	1.570	1.562	1.236	1.518
	[1.431 - 2.169]	[1.305 - 2.542]	[1.003 - 1.719]	[1.044 - 2.403]	[0.997 - 1.711]	[1.022 - 2.359]	[0.997 - 1.711]	[1.033 - 2.386]	[1.029 - 2.373]	[0.944 - 1.618]	[1.000 - 2.306]
Mother´s lifetime earning rank											
First quintile	1.128	1.237	1.035	1.096	1.023	1.079	1.024	1.079	1.081	1.033	1.076
	[1.057 - 1.204]	[1.101 - 1.390]	[0.967 - 1.107]	[0.970 - 1.239]	[0.956 - 1.095]	[0.954 - 1.219]	[0.957 - 1.096]	[0.955 - 1.220]	[0.956 - 1.222]	[0.966 - 1.107]	[0.951 - 1.217]

Second quintile	1.087	1.083	1.058	1.037	1.055	1.035	1.055	1.038	1.039	1.068	1.049
	[1.036 - 1.140]	[0.993 - 1.182]	[1.007 - 1.110]	[0.950 - 1.133]	[1.005 - 1.107]	[0.948 - 1.131]	[1.005 - 1.108]	[0.868 - 1.043]	[0.951 - 1.135]	[1.018 - 1.122]	[0.960 - 1.146]
Third quintile **[Ref]**	**1*****	**1*****	**1***	**1***	**1***	**1***	**1***	**1***	**1***	**1****	**1***
Fourth quintile	0.993	0.923	1.011	0.951	1.012	0.953	1.011	0.957	0.957	0.991	0.943
	[0.944 - 1.045]	[0.839 - 1.014]	[0.960 - 1.064]	[0.864 - 1.046]	[0.961 - 1.065]	[0.869 - 1.050]	[0.960 - 1.064]	[0.870 - 1.053]	[0.870 - 1.053]	[0.941 - 1.044]	[0.857 - 1.038]
Fifth quintile	0.957	0.924	0.998	0.991	0.998	0.993	0.997	1.001	1.000	0.953	0.963
	[0.895 - 1.022]	[0.816 - 1.046]	[0.931 - 1.069]	[0.871 - 1.128]	[0.931 - 1.070]	[0.873 - 1.130]	[0.931 - 1.069]	[0.880 - 1.139]	[0.879 - 1.137]	[0.889 - 1.022]	[0.846 - 1.096]
Father´s lifetime earning rank											
First quintile	1.212	1.306	1.064	1.114	1.057	1.110	1.054	1.091	1.088	1.033	1.057
	[1.139 - 1.288]	[1.170 - 1.458]	[0.997 - 1.135]	[0.991 - 1.252]	[0.994 - 1.130]	[0.988 - 1.248]	[0.988 - 1.124]	[0.970 - 1.226]	[0.968 - 1.233]	[0.968 - 1.102]	[0.940 - 1.188]
Second quintile	1.018	1.014	0.983	0.968	0.982	0.965	0.980	0.951	0.950	0.979	0.943
	[0.969 - 1.070]	[0.926 - 1.111]	[0.935 - 1.033]	[0.883 - 1.062]	[0.934 - 1.032]	[0.880 - 1.058]	[0.932 - 1.031]	[0.868 - 1.043]	[0.867 - 1.042]	[0.931 - 1.030]	[0.860 - 1.034]
Third quintile **[Ref]**	**1*****	**1*****	**1****	**1***	**1***	**1***	**1****	**1***	**1***	**1****	**1***
Fourth quintile	0.910	0.890	0.929	0.919	0.933	0.924	0.935	0.937	0.938	0.922	0.930
	[0.864 - 0.960]	[0.808 - 0.980]	[0.881 - 0.980]	[0.833 - 1.014]	[0.893 - 0.993]	[0.838 - 1.019]	[0.886 - 0.986]	[0.850 - 1.034]	[0.851 - 1.035]	[0.874 - 0.973]	[0.843 - 1.026]
Fifth quintile	0.905	0.882	0.940	0.948	0.946	0.954	0.948	0.984	0.985	0.914	0.961
	[0.851 - 0.962]	[0.785 – 0.989]	[0.881 - 1.003]	[0.839 - 1.071]	[0.899 - 1.023]	[0.844 - 1.078]	[0.888 - 1.012]	[0.871 - 1.112]	[0.872 - 1.113]	[0.856 - 0.976]	[0.850 - 1.087]

Intergenerational biological variables

PCOS in mother											
No **[Ref]**	**1*****	**1*****	**1*****	**1*****	**1*****	**1*****	**1*****	**1*****	**1*****	**1*****	**1*****
Yes	2.975	3.693	2.818	3.360	2.775	3.297	2.795	3.117	3.150	2.612	2.970
	[2.433 – 3.637]	[2.671 – 5.106]	[2.305 - 3.445]	[2.429- 4.648]	[2.269 - 3.393]	[2.383 - 4.563]	[2.286 - 3.418]	[2.291 - 4.353]	[2.275 - 4.360]	[2.132 - 3.320]	[2.143 - 4.116]
DM in mother											
No **[Ref]**	**1*****	**1*****	**1*****	**1*****	**1*****	**1*****	**1*****	**1*****	**1*****	**1*****	**1*****
Yes	1.505	1.757	1.474	1.694	1.407	1.589	1.401	1.378	1.370	1.377	1.341
	[1.389 – 1.631]	[1.497 – 2.062]	[1.360 - 1.598]	[1.443 - 1.989]	[1.297 - 1.526]	[1.351 - 1.867]	[1.292 - 1.520]	[1.161 - 1.611]	[1.162 - 1.616]	[1.270 - 1.494]	[1.137 - 1.581]
PCOS in sister											
Sister without PCOS **[Ref]**	**1*****	**1*****	**1*****	**1*****	**1*****	**1*****	**1*****	**1*****	**1*****	**1*****	**1*****

No sister	1.119	1.126	1.114	1.120	1.103	1.119	1.103	1.108	1.108	1.100	1.105
	[1.077 - 1.170]	[1.051 - 1.205]	[1.072 - 1.157]	[1.046 - 1.199]	[1.060 - 1.147]	[1.043 - 1.201]	[1.061 - 1.148]	[1.033 - 1.190]	[1.032 - 1.190]	[1.057 - 1.144]	[1.030 - 1.186]
Sister with PCOS	5.210	5.506	5.148	5.377	4.970	5.143	4.957	5.003	5.002	4.739	4.786
	[4.708 - 5.765]	[4.610 - 6.576]	[4.652 - 5.697]	[4.501 - 6.424]	[4.490 - 5.502]	[4.301 - 6.149]	[4.479 - 5.487]	[4.183 – 5.983]	[4.183 – 5.982]	[4.281 - 5.245]	[4.002 - 5.724]
**Early-life factors - before birth**											
Mother´s smoking in early pregnancy[a]											
Not smoking **[Ref]**	–	**1*****	–	**1*****			–	**1*****	**1*****	–	**1*****
Moderate smoker	–	1.170	–	1.172			–	1.168	1.162	–	1.146
		[1.073 - 1.276]		[1.075 - 1.279]				[1.069 - 1.277]	[1.062 - 1.270]		[1.047 - 1.253]
Heavy smoker	–	1.301	–	1.282			–	1.260	1.250	–	1.221
		[1.176 - 1.440]		[1.158 - 1.419]				[1.135 - 1.399]	[1.125 - 1.390]		[1.098 - 1.358]
Weight gain during pregnancy[a]											
Inadequate	–	1.016	–	1.046			–	1.041	1.034	–	1.037
		[0.932 - 1.107]		[0.959 - 1.140]				[0.954 - 1.135]	[0.947 - 1.128]		[0.950 - 1.132]
Appropriate **[Ref]**	–	**1****	–	**1***			–	**1***	**1***	–	**1***
Excessive	–	1.139	–	1.057			–	1.047	1.051	–	1.047
		[1.053 - 1.231]		[0.976 - 1.146]				[0.966 - 1.134]	[0.969 - 1.139]		[0.966 - 1.136]
Mother´s BMI at the beginning of pregnancy[a]											
Underweight	–	0.904	–	0.893			–	0.878	0.873	–	0.866
		[0.788 - 1.037]		[0.777 - 1.025]				[0.765 - 1.009]	[0.760 - 1.003]		[0.754 - 0.995]
Normal **[Ref]**	–	**1*****	–	**1*****			–	**1*****	**1*****	–	**1*****
Overweight	–	1.488	–	1.470			–	1.397	1.394	–	1.396
		[1.361 - 1.628]		[1.340 - 1.612]				[1.271 - 1.494]	[1.268 - 1.532]		[1.270 - 1.534]
Obese	–	1.902	–	1.871			–	1.698	1.694	–	1.681
		[1.617 - 2.361]		[1.590 - 2.202]				[1.438 - 2.005]	[1.434 - 2.001]		[1.423 - 1.986]
**Early-life factors - outcomes**											
Birth weight, grams[b]											

Less than 2 500	1.095	0.966	1.092	0.851			1.072		0.821	1.071	0.822
	[0.989 - 1.213]	[0.782 - 1.193]	[0.970 - 1.230]	[0.669 - 1.082]			[0.952 - 1.207]		[0.760 - 1.003]	[0.951 - 1.206]	[0.647 - 1.045]
2 500 – 2 999	1.129	1.102	1.130	1.073			1.109		1.051	1.106	1.049
	[1.066 - 1.195]	[0.995 - 1.225]	[1.066 - 1.197]	[0.963 - 1.195]			[1.046 - 1.176]		[0.942 - 1.172]	[1.043 - 1.172]	[0.941 - 1.170]
3 000 – 3 499 **[Ref]**	**1*** **	**1***	**1*** **	**1***			**1** **		**1***	**1** **	**1***
3 500 – 3 999	0.992	0.932	0.983	0.932			0.996		0.940	1.001	0.944
	[0.949 - 1.037]	[0.860 - 1.010]	[0.940 - 1.027]	[0.860 - 1.011]			[0.953 - 1.041]		[0.866 - 1.020]	[0.957 - 1.046]	[0.869 - 1.025]
4 000 – 4 499	1.056	0.984	1.034	0.977			1.053		0.967	1.063	0.972
	[0.994 - 1.122]	[0.880 - 1.100]	[0.972 - 1.099]	[0.873 - 1.093]			[0.990 - 1.120]		[0.862 - 1.086]	[0.999 - 1.131]	[0.866 - 1.092]
Fetal macrosomia, over 4 500	1.071	1.182	1.033	1.162			1.040		1.105	1.056	1.125
	[0.942 - 1.218]	[0.950 - 1.470]	[0.908 - 1.175]	[0.933 - 1.447]			[0.914 - 1.183]		[0.884 - 1.382]	[0.928 - 1.202]	[0.900 - 1.407]
One-minute Apgar											
Less than or equal to 7	1.184	1.285	1.163	1.263			1.35		1.189	1.122	1.207
	[1.086 - 1.291]	[1.086 - 1.521]	[1.065 - 1.269]	[1.066 - 1.497]			[1.039 - 1.239]		[1.003 - 1.411]	[1.027 - 1.225]	[1.016 - 1.433]
8	1.132	1.067	1.119	1.055			1.102		1.017	1.093	1.036
	[1.045 - 1.227]	[0.912 - 1.249]	[1.032 - 1.213]	[0.901 - 1.235]			[1.017 - 1.195]		[0.868 - 1.192]	[1.008 - 1.192]	[0.883 - 1.215]
9	1.127	1.180	1.124	1.176			1.121		1.162	1.124	1.200
	[1.068 - 1.190]	[1.059 - 1.315]	[1.064 - 1.187]	[1.055 - 1.310]			[1.062 - 1.184]		[1.042 - 1.295]	[1.042 - 1.295]	[1.073- 1.341]
10 **[Ref]**	**1*** **	**1** **	**1*** **	**1** **			**1*** **		**1** **	**1** **	**1** **
Gestational age, weeks											
Extremely preterm, less than	0.895	0.820	0.820	0.856			0.797		0.837	0.797	0.846
28	[0.427 - 1.879]	[0.115 – 5.822]	[0.388 - 1.732]	[0.120 – 6.129]			[0.377 - 1.684]		[0.117 - 5.994]	[0.377 - 1.683]	[0.118 – 6.058]
Very preterm, between 28-32	0.991	1.027	0.904	1.124			0.893		1.138	0.892	1.153
	[0.769 - 1.277]	[0.596 - 1.771]	[0.688 - 1.188]	[0.631 – 2.004]			[0.680 - 1.172]		[0.638 – 2.027]	[0.679 - 1.171]	[0.647 – 2.052]
Moderate to late preterm, 33-	1.126	1.290	1.044	1.287			1.033		1.274	1.032	1.273
36	[1.023 - 1.239]	[1.096 - 1.517]	[0.939 - 1.162]	[1.075 - 1.541]			[0.928 - 1.150]		[1.065 - 1.524]	[0.928 - 1.148]	[1.064- 1.522]
Full term, 37 -41 **[Ref]**	**1*** **	**1***	**1*** **	**1***			**1*** **		**1***	**1*** **	**1***
Post-term, 42 or over	1.191	1.125	1.199	1.128			1.195		1.110	1.187	1.100
	[1.126 - 1.260]	[0.983 - 1.288]	[1.133 - 1.269]	[0.984 - 1.293]			[1.129 - 1.265]		[0.968 - 1.273]	[1.121 - 1.257]	[0.959 - 1.261]

Individual adult life factors – index woman

Educational attainment

Under primary	1.148	1.148	1.144	1.139					1.109	1.063
	[0.875 - 1.506]	[0.754 - 1.747]	[0.872 - 1.500]	[0.749 - 1.733]					[0.847 - 1.452]	[0.701 - 1.613]
Primary school	1.288	1.368	1.276	1.349					1.221	1.243
	[1.201 - 1.382]	[1.211 - 1.545]	[1.190 - 1.369]	[1.195 - 1.523]					[1.137 - 1.310]	[1.100 - 1.405]
Secondary school **[Ref]**	**1*****	**1*****	**1*****	**1*****					**1*****	**1****
University	1.033	0.986	1.014	0.982					1.063	1.051
	[0.989 - 1.079]	[0.905 - 1.074]	[0.970 - 1.059]	[0.901 - 1.071]					[1.015 - 1.112]	[0.962 - 1.149]

Civil status

Not married, not registered re-	1.261	1.596	1.274	1.587					1.242	1.459
lationship	[1.192 - 1.333]	[1.388 - 1.835]	[1.205 - 1.347]	[1.380 - 1.824]					[1.175 - 1.313]	[1.268 - 1.678]
Married, registered relation-	**1*****	**1*****	**1*****	**1*****					**1*****	**1*****
ship **[Ref]**										

Abbreviations: BMI, body mass index; CI, confidence interval; DM, diabetes mellitus; HR, hazard ratio; PCOS, polycystic ovary syndrome; Ref, reference; Res, restricted.
Full: analyses were done on the full sample; women born between 1973 and 1995 [n = 977 637].
Restricted.: analyses were done on the restricted sample; women born between 1982 and 1995 [n = 302 638].
[a]Available for a subgroup of women born between 1982 and 1995 [n = 302 638].
[b]No significant nonlinearity was detected when testing for birth weight and its quadratic term.
Model 1: Minimally adjusted analyses that only adjust for the given variable and birth year.
Model 2: Further adjusted models additionally adjust for variables under the same category, for instance Intergenerational biological variables: OD in mother, DM in mother, OD in sister, with additional adjustment for birth year.
Model 3: Model includes all Intergenerational sociodemographic and biological variables with additional adjustment for birth year.
Model 4: Model includes all Intergenerational sociodemographic and biological variables, and Early-life factors with additional adjustment for birth year.
Model 5: Model includes all Intergenerational sociodemographic and biological variables, Early-life factors and Individual adult life factors with additional adjustment for birth year and counties.
***p<0.001
**p<0.01
*p<0.5

Table S3. Associations of social and biological characteristics with rate of polycystic ovary syndrome with age restriction among Swedish women born between 1973-1995

	**Sensitivity analysis:** **Age <20yrs** n = 644 692	**Sensitivity analysis:** **Age <25yrs** n = 803 948	**Full Model:** **Follow-up 1973-1995** n = 977 637
**Predictor**	**HR [CI 95%]**	**HR [CI 95%]**	**HR [CI 95%]**
	**Intergenerational sociodemographic variables**		
Birth order			
First born **[Ref]**	**1*** **	**1*** **	**1** **
Second born	0.892	0.908	0.924
	[0.842 - 0.944]	[0.867 - 0.951]	[0.884 - 0.965]
Third born or higher	0.938	0.947	0.957
	[0.873 - 1.008]	[0.887 - 1.010]	[0.898 - 1.020]
Mother´s age at index woman´s birth, years			
Less than or equal to 18	1.091	1.084	1.118
	[0.849 - 1.402]	[0.894 - 1.313]	[0.964 - 1.296]
Between 19-35 **[Ref]**	**1***	**1***	**1***
Greater than 35	1.053	1.040	1.036
	[0.969 - 1.144]	[0.967 - 1.118]	[0.969 - 1.109]
Mother´s educational attainment			
Primary	0.984	0.978	0.987
	[0.911 - 1.063]	[0.918 - 1.042]	[0.935 - 1.041]
Secondary **[Ref]**	**1***	**1**	**1**
University	0.953	0.976	0.981
	[0.897 - 1.012]	[0.927 - 1.026]	[0.937 - 1.027]
Father´s educational attainment			
Primary	1.020	1.036	1.018

	[0.957 - 1.086]	[0.983 - 1.092]	[0.972 - 1.066]
Secondary **[Ref]**	**1**	**1***	**1***
University	0.978	0.991	0.977
	[0.916 - 1.045]	[0.938 - 1.047]	[0.930 - 1.026]
Mother´s country of birth			
Sweden **[Ref]**	**1*****	**1*****	**1*****
Nordics, excluding Sweden	0.981	0.959	0.988
	[0.861 - 1.117]	[0.860 - 1.068]	[0.900 - 1.086]
Europe, North America and Oceania	1.340	1.329	1.299
	[1.175 - 1.529]	[1.188 - 1.487]	[1.173 - 1.438]
Africa	1.295	1.267	1.211
	[0.969 - 1.731]	[0.967 - 1.661]	[0.933 - 1.572]
Asia	1.527	1.541	1.553
	[1.262 - 1.848]	[1.294 - 1.836]	[1.313 - 1.838]
South America	1.406	1.368	1.480
	[1.016 - 1.947]	[1.012 - 1.848]	[1.113 - 1.968]
Father´s country of birth			
Sweden **[Ref]**	**1*****	**1*****	**1*****
Nordics, excluding Sweden	1.037	1.052	1.047
	[0.901 - 1.192]	[0.936 - 1.182]	[0.945 - 1.160]
Europe, North America and Oceania	1.188	1.153	1.134
	[1.046 - 1.349]	[1.035 - 1.284]	[1.030 - 1.248]
Africa	1.169	1.179	1.237
	[0.907 - 1.507]	[0.939 - 1.480]	[1.001 - 1.528]
Asia	1.547	1.554	1.516
	[1.283 - 1.866]	[1.310 - 1.844]	[1.287 - 1.787]
South America	1.254	1.281	1.236
	[0.924 - 1.702]	[0.969 - 1.695]	[0.944 - 1.618]

Mother´s lifetime earning rank			
First quintile	1.054	1.027	1.033
	[0.962 - 1.156]	[0.951 - 1.110]	[0.966 - 1.107]
Second quintile	1.110	1.080	1.068
	[1.040 - 1.185]	[1.022 - 1.141]	[1.018 - 1.122]
Third quintile **[Ref]**	**1*** **	**1** **	**1** **
Fourth quintile	0.969	0.974	0.991
	[0.903 - 1.039]	[0.919 - 1.033]	[0.941 - 1.044]
Fifth quintile	0.941	0.962	0.953
	[0.855 - 1.035]	[0.890 - 1.040]	[0.889 - 1.022]
Father´s lifetime earning rank			
First quintile	1.061	1.015	1.033
	[0.974 - 1.156]	[0.944 - 1.093]	[0.968 - 1.102]
Second quintile	0.988	0.976	0.979
	[0.923 - 1.057]	[0.922 - 1.033]	[0.931 - 1.030]
Third quintile **[Ref]**	**1***	**1***	**1** **
Fourth quintile	0.946	0.933	0.922
	[0.880 - 1.018]	[0.879 - 0.991]	[0.874 - 0.973]
Fifth quintile	0.902	0.904	0.914
	[0.823 - 0.988]	[0.839 - 0.974]	[0.856 - 0.976]
	**Intergenerational biological variables**		
PCOS in mother			
No **[Ref]**	**1*** **	**1*** **	**1*** **
Yes	3.247	2.900	2.612
	[2.578 - 4.089]	[2.344 - 3.587]	[2.132 - 3.320]
DM in mother			
No **[Ref]**	**1*** **	**1*** **	**1*** **

Yes	1.400	1.400	1.377
	[1.241 - 1.578]	[1.271 - 1.541]	[1.270 - 1.494]
PCOS in sister			
Sister without PCOS **[Ref]**	**1*** **	**1*** **	**1*** **
No sister	1.133	1.100	1.100
	[1.075 - 1.194]	[1.053 - 1.149]	[1.057 - 1.144]
Sister with PCOS	5.270	4.687	4.739
	[4.633 - 5.995]	[4.194 - 5.238]	[4.281 - 5.245]
		**Early-life factors - outcomes**	
Birth weight, grams			
Less than 2 500	0.976	1.019	1.071
	[0.831 - 1.147]	[0.890 - 1.168]	[0.951 - 1.206]
2 500 – 2 999	1.058	1.071	1.106
	[0.977 - 1.145]	[1.002 - 1.145]	[1.043 - 1.172]
3 000 – 3 499 **[Ref]**	**1***	**1***	**1****
3 500 – 3 999	0.976	1.001	1.001
	[0.919 - 1.037]	[0.953 - 1.053]	[0.957 - 1.046]
4 000 – 4 499	1.037	1.051	1.063
	[0.954 - 1.128]	[0.980 - 1.126]	[0.999 - 1.131]
Fetal macrosomia, over 4 500	1.153	1.063	1.056
	[0.977 - 1.362]	[0.920 - 1.228]	[0.928 - 1.202]
One-minute Apgar			
Less than or equal to 7	1.203	1.166	1.122
	[1.057 - 1.368]	[1.045 - 1.301]	[1.027 - 1.225]
8	1.133	1.128	1.093
	[1.007 - 1.276]	[1.020 - 1.248]	[1.008 - 1.192]
9	1.183	1.158	1.124

	[1.085 - 1.290]	[1.075 - 1.248]	[1.042 - 1.295]
10 **[Ref]**	**1*** **	**1*** **	**1** **
Gestational age, weeks			
Extremely preterm, less than 28	0.642	0.791	0.797
	[0.238 - 1.731]	[0.352 - 1.778]	[0.377 - 1.683]
Very preterm, between 28-32	1.018	0.940	0.892
	[0.724 - 1.432]	[0.695 - 1.270]	[0.679 - 1.171]
Moderate to late preterm, 33-36	1.106	1.079	1.032
	[0.971 - 1.260]	[0.962 - 1.212]	[0.928 - 1.148]
Full term, 37 -41 **[Ref]**	**1***	**1****	**1*****
Post-term, 42 or over	1.037	1.126	1.187
	[0.935 - 1.151]	[1.048 - 1.209]	[1.121 - 1.257]
	**Individual adult life factors -index woman**		
Educational attainment			
Under primary	1.079	1.095	1.109
	[0.820 - 1.421]	[0.834 - 1.437]	[0.847 - 1.452]
Primary school	1.189	1.205	1.221
	[1.087 - 1.301]	[1.114 - 1.304]	[1.137 - 1.310]
Secondary school **[Ref]**	**1****	**1*****	**1*****
University	1.010	1.033	1.063
	[0.943 - 1.082]	[0.979 - 1.090]	[1.015 - 1.112]
Civil status			
Not married, not registered relationship	1.451	1.340	1.242
	[1.300 - 1.619]	[1.247 - 1.440]	[1.175 - 1.313]
Married, registered relationship **[Ref]**	**1*****	**1*****	**1*****

***p<0.001
**p<0.01

*p<0.5

Figure S1. Flow diagram of the study sample

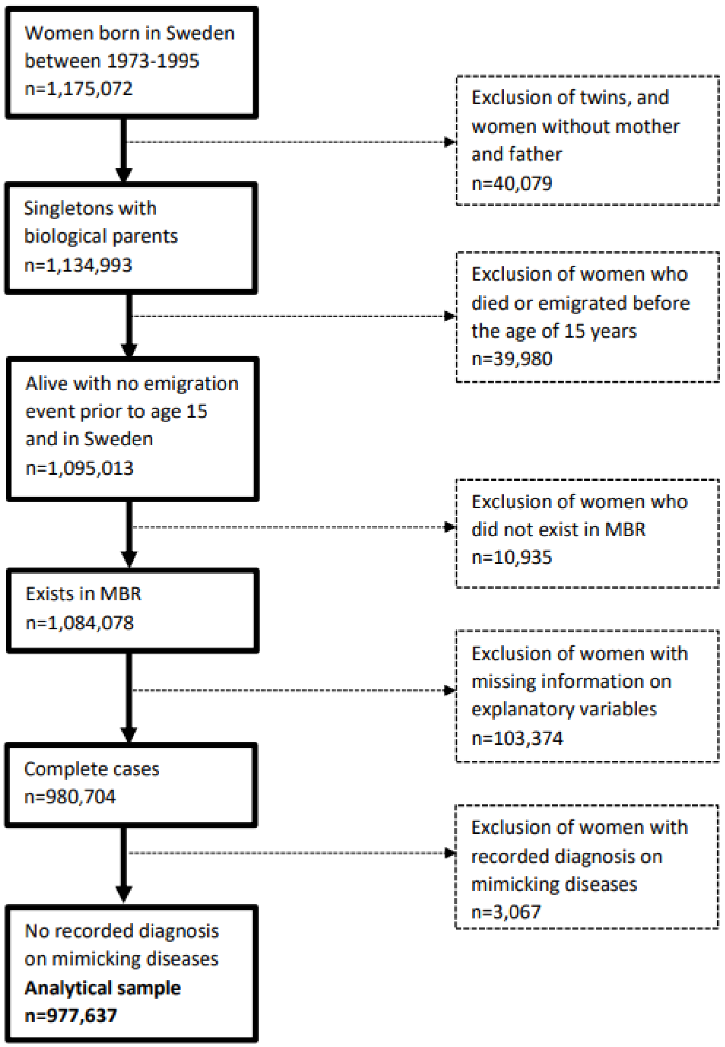

Figure S2. Conceptual framework depicting explanatory variables that capture pre-, peri-, and postnatal context of polycystic ovary syndrome

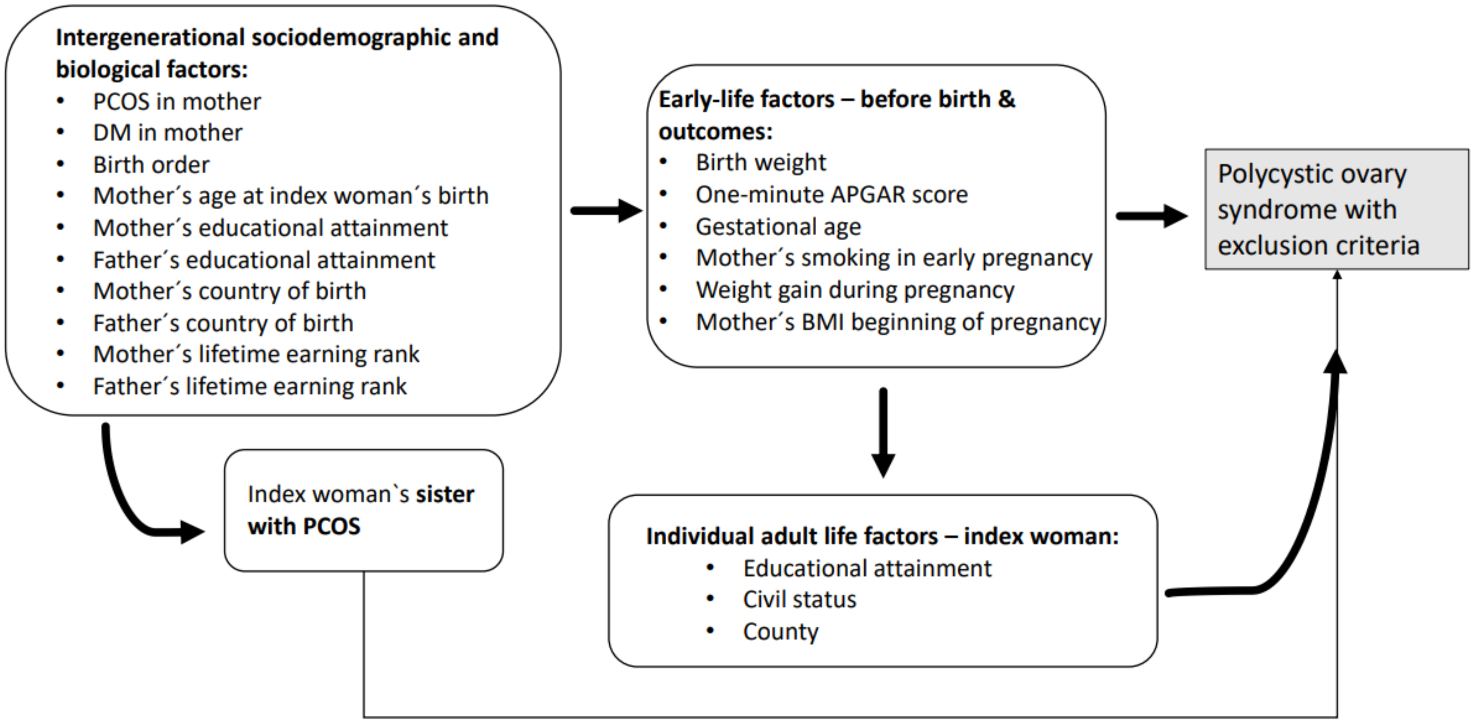

Supplement: Supplementary file 1 [file ijerph-20-07083-s001.zip › ijerph-2647008-supplementary.pdf]
